# Limb bone histology records birth in mammals

**DOI:** 10.1371/journal.pone.0198511

**Published:** 2018-06-20

**Authors:** Carmen Nacarino-Meneses, Meike Köhler

**Affiliations:** 1 Department of Evolutionary Paleobiology, Institut Català de Paleontologia Miquel Crusafont (ICP), Campus de la Universitat Autònoma de Barcelona, Bellaterra, Barcelona, Spain; 2 ICREA, Barcelona, Spain; Universidade Federal de Sao Paulo, BRAZIL

## Abstract

The annual cyclicality of cortical bone growth marks (BGMs) allows reconstruction of some important life history traits, such as longevity, growth rate or age at maturity. Little attention has been paid, however, to non-cyclical BGMs, though some record key life history events such as hatching (egg-laying vertebrates), metamorphosis (amphibians), or weaning (suggested for *Microcebus* and the hedgehog). Here, we investigate the relationship between non-cyclical BGMs and a stressful biological event in mammals: the moment of birth. In the present study, we histologically examine ontogenetic series of femora, tibiae and metapodia in several extant representatives of the genus *Equus* (*E*. *hemionus*, *E*. *quagga* and *E*. *grevyi*). Our analysis reveals the presence of a non-cyclical growth mark that is deposited around the moment of birth, analogous to the neonatal line described for teeth. We therefore refer to it as neonatal line. The presence of this feature within the bone cross-section agrees with a period of growth arrest in newborn foals regulated by the endocrine system. The neonatal line is accompanied by modifications in bone tissue type and vascularization, and has been identified in all bones studied and at different ontogenetic ages. Our discovery of a non-cyclical BGM related to the moment of birth in mammals is an important step towards the histological reconstruction of life histories in extant and fossil equids.

## Introduction

The study of bone microstructure provides key insights into the life history strategy of extant and extinct vertebrates. Bones present distinctive histological features that allow inference of important life history traits of species, such as growth rate [1–4], longevity [5–8] or age at maturity [9–13]. The arrangement of collagen fibers within the bone matrix, its vascularization and the density and number of bone cells are directly related to the rate of bone deposition [1,3,14–17]. Analysis of these histological characteristics, hence, allows estimation of growth rate in extant and extinct taxa [2,4,6,10,18,19]. Inferences of longevity and age at maturity, on the other hand, are based on the study of bone growth marks (BGMs) by means of skeletochronology [6,9–12,20,21]. This sort of studies relies on the annual periodicity of cyclical growth marks (CGMs), which record hormonal and physiological cycles [22] that are synchronized with seasonal photoperiod and resource availability [15,23].

However, not all BGMs reflect periodical growth [20,23,24]. Although the ultimate causes of deposition of non-cyclical BGMs are poorly understood, they are supposed to record stressful biological events that affect the organism [24] and thereby periosteal bone growth [20]. During their growth, long bones increase in diameter and change in shape to cope with biomechanical loads [25,26]. Microscopically, these diaphyseal changes involve the simultaneous deposition of bone at the periosteal (external) surface and its resorption at the endosteal (internal) margin of the bone shaft [15,20,25]. This process, named cortical drift [15,25], may leave non-cyclical resorption lines within the bone cross-section [20]. These consist of incomplete circles that are limited to the affected bone area [20]. Other non-cyclical BGMs have been related to specific biological events that represent moments of physiological stress in the individual [23,24]. These BGMs do not result from resorption but they are deposited along the whole growth front of the bone, thus forming complete circles within the bone cross-section at the time of deposition [23]. Several authors have described non-cyclical BGMs that record hatching in amphibians and reptiles [27–30], metamorphosis in amphibians [31–38] and weaning in mammals [5,24,39].

In a previous study of limb bone histology of the Asiatic wild ass (*Equus hemionus*), we identified a non-cyclical BGM in most of the bone cortices analyzed [11]. On that basis, we develop the present research, which primary objective is to thoroughly explore the causes that led to the deposition of this feature in *Equus*, and to increase our understanding of the appearance of non-cyclical BGMs of physiological origin in mammals in general. Thus, we aim to investigate the non-cyclical BGM previously found in *E*. *hemionus* and to explore its presence in other *Equus* species at different ontogenetic stages from perinates to adults. We will further examine if this non-cyclical BGM appears associated to other histological changes. When deposited as a consequence of a physiologically stressful event, such as hatching, these structures have been described to be associated with changes in bone tissue type and vascularization [27,29,30].

## Material and methods

Thin sections of femur, tibia, metacarpus and metatarsus were prepared from ontogenetic series (perinates to adults) of *E*. *hemionus*, *E*. *quagga* (Plains zebra) and *E*. *grevyi* (Grevy’s zebra) (Table 1). We selected these *Equus* species because they cover almost all habitats and life histories of the genus [40–44]. We studied 9 individuals of *E*. *hemionus*, 11 of *E*. *quagga* and 4 of *E*. *grevyi* (Table 1). The sample comprises 35 thin sections of *E*. *hemionus*, 14 of *E*. *quagga* and 10 of *E*. *grevyi*, totaling 59 the thin sections of the study (Table 1). As shown in Table 1, specimens differ in habitat, age and sex. Most specimens of this study lived captive in the Hagenbeck Zoo (Hamburg, Germany) and are stored at the Zoological Institute of Hamburg University (Germany). Several zebra specimens (Table 1) lived semi-captive in the African Reserve of Sigean (Sigean, France) and belong to the collections of the Catalan Institute of Paleontology (Barcelona, Spain). Finally, adult individuals of Asiatic wild ass lived wild in the Gobi Desert (Table 1). Found killed by poachers, they were collected during the Mongolian-German Biological Expeditions (2001–2006) [45] and are currently housed at the Museum of Domesticated Animals (Halle, Germany). Curators and veterinarians of the different institutions provided information of the sex (R. Schafberg and B. Lamglait, pers. comm.). Additionally, B. Lamglait of the African Reserve of Sigean (France) facilitated the exact age at death of the animals that lived in their institution (B. Lamglait, pers. comm.) and R. Schafberg provided an estimated age for adult kulans (R. Schafberg, pers. comm.). The rest of specimens belonging to museum collections were aged according to the eruption and wear pattern of the species [46–50]. In adult individuals, these estimations were validated by the number of annual cementum layers present in the first lower incisor [46,50]. In several subadult specimens, age calculation was also corroborated by crown formation time inferred from the study of enamel laminations [50]. We grouped our specimens into the classical life stages proposed for equids (Table 1): foals (less than 1 year), yearlings (between 1 and 2 years), juveniles (between 2 and 4 years) and adults (more than 4 years). We further considered an additional age category for the youngest animals of our sample: the perinatal stage. Perinatal specimens were aged by the species-specific tooth eruption sequence, which starts between the first (in *E*. *quagga*; [47]) and the third week of life (in *E*. *hemionus*; [46]). Tooth eruption in our animals indicates that they were younger than 1 and 3 weeks respectively, but we cannot assess from this methodology whether they died during birth, just after birth or few days before birth. Therefore, we preferred to use the term “perinate” to designate this age group, instead of “newborn”, “fetus” or similar.

**Table 1:** Sample studied.

Species	Code	Collection code	Age	Age group	Sex	Habitat	Bones studied	Collection
***E*. *hemionus***	IPS83152	UH 6970	< 3 w.	Perinate	-	HZ	Fe, Ti, Mc, Mt	ZIHU
	IPS83153	UH 7009	5 mo.	Foal	M	HZ	Fe, Ti, Mc, Mt	ZIHU
	IPS83154	UH 7016	5 mo.	Foal	M	HZ	Fe, Ti, Mc	ZIHU
	IPS83151	UH 5929	6 mo.	Foal	-	HZ	Fe, Ti, Mc, Mt	ZIHU
	IPS83150	UH 5547	6–12 mo.	Yearling	-	HZ	Fe, Ti, Mc, Mt	ZIHU
	IPS83149	UH 5546	6–12 mo.	Yearling	-	HZ	Fe, Ti, Mc, Mt	ZIHU
	IPS83155	UH 7528	1–2 y.	Juvenile	F	HZ	Fe, Ti, Mc, Mt	ZIHU
	IPS83876	225	4.5 y.	Adult	F	GD	Fe, Ti, Mc, Mt	MDA
	IPS83877	381	8 y.	Adult	M	GD	Fe, Ti, Mc, Mt	MDA
***E*. *quagga***	IPS92343	UH 903	< 1 w.	Perinate	-	HZ	Fe	ZIHU
	IPS92344	UH 6189	< 1 w.	Perinate	-	HZ	Fe	ZIHU
	IPS101798	A15/007	4 mo.	Foal	F	ARS	Fe, Ti	ICP
	IPS92345	UH 7467	4 mo.	Foal	-	HZ	Fe	ZIHU
	IPS92342	UH 7529	5 mo.	Foal	F	HZ	Fe	ZIHU
	IPS104356	A16/085	9 mo.	Foal	F	ARS	Ti, Mc, Mt	ICP
	IPS92341	UH 800	1 y.	Yearling	-	HZ	Fe	ZIHU
	IPS104357	A15/013	1 y.	Yearling	F	ARS	Ti	ICP
	IPS101800	A14/330	1 y. + 2mo.	Yearling	M	ARS	Fe	ICP
	IPS101801	A15/049	1 y. + 2mo.	Yearling	F	ARS	Ti	ICP
	IPS92346	UH 397	5 y.	Adult	-	HZ	Fe	ZIHU
***E*. *grevyi***	IPS101802	A15/020	1 mo.	Foal	M	ARS	Fe, Ti	ICP
	IPS84964	UH 8255	4 mo.	Foal	F	HZ	Fe, Ti, Mt	ZIHU
	IPS101804	A15/120	2 y.	Juvenile	M	ARS	Fe, Ti	ICP
	IPS84963	UH 7111	5 y.	Adult	F	HZ	Fe, Ti, Mt	ZIHU

w.: weeks; mo.: months; y.: years; M: male; F: female; Fe: femur; Ti: tibia; Mc: metacarpus; Mt: metatarsus; GD: Gobi Desert (Mongolia, Asia); HZ: Hagenbeck Zoo (Hamburg, Germany); ARS: African Reserve of Sigean (Sigean, France); ICP: Catalan Institute of Paleontology (Barcelona, Spain); MDA: Museum of Domesticated Animals (Halle, Germany); ZIHU: Zoological Institute of Hamburg University (Hamburg, Germany).

Histological slices were prepared following standard methods in our laboratory [7,11]. From the mid-shaft of each bone, we extracted a block of 3 cm that was then embedded in epoxy resin (Araldite 2020). This block was later cut into two halves using a low speed diamond saw (IsoMet, Buehler). The cut surfaces were then polished with carborundum powder or with a MetaServ 250 (Buehler) and fixed to a frosted glass with Loctite 358, an ultraviolet-curing adhesive. Samples were then cut and grounded with a diamond saw (PetroThin, Buehler) and polished again either with carborundum powder or with a grinder-polisher (MetaServ 250, Buehler) to obtain a final thickness of approximately 100–120 μm. Finally, thin sections were covered with a mix of oils [51] to improve the visualization of the histological features under the microscope. All thin sections obtained in the present research belong to the collections of the Catalan Institute of Paleontology (ICP, Barcelona, Spain) and are available to researchers. Histological thin sections were observed using polarized light under a Leica DM 2500P microscope and under a Zeiss Scope.A1 microscope. Micrographs were taken with the incorporated cameras (Leica DFC490 and AxioCam ICc5). Several samples were also analyzed under polarized light with a ¼ λ filter to facilitate the identification of the bone tissue types and the visualization of the BGMs [52].

We recorded the presence or absence of non-cyclical BGMs in all species, bones and ontogenetic stages under study. The classification of BGMs as cyclical or non-cyclical was made considering the position of the BGM within the bone cross-section in relation to the age of the individual. For instance, a BGM identified in the mid-cortex of a kulan foal aged 5 months (e.g. IPS83153, Table 1) was considered as non-cyclical [11]. As *E*. *hemionus* tends to give birth in summer [40,53], the CGM that records the growth arrest during the unfavorable season (i.e. winter for this species) [22] is expected to be found in its outermost cortex. Accordingly, any BGM deposited in the internal part of the cross-section at this ontogenetic stage should be considered as non-cyclical. In yearlings, the identification of non-cyclical BGMs and its differentiation from cyclical ones was made following a similar procedure as in foals, that is, considering the age of the individual and the location of the BGM within the bone cortex. In yearlings, we identified an additional BGM to that expected from age (2 BGMs in total) [11]. Because CGMs are known to be deposited annually [22] and these specimens are aged around one year (Table 1), we considered the most external BGM as a CGM and the most internal BGM as a non-cyclical one [11]. In juveniles and adults, we did not always find an extra BGM in relation to their age, but non-cyclical features were recognized by performing superimposition of individuals [20]. In these age categories, those BGMs were considered as non-cyclical whether they were deposited earlier than the CGM found in yearlings [11]. Superimposition [20] was also applied to identify non-cyclical BGMs that have been erased by resorption of the medullary cavity. Likewise, this technique was employed to evaluate the correspondence between the perimeter of the non-cyclical BGM identified in different ontogenetic stages and the perimeter of the perinatal individual, with the objective to find a correlation between this non-cyclical feature and a specific key life history event such as the moment of birth. In our sample, we have a complete set of all limb bones (femur, tibia, metacarpus and metatarsus) of the perinatal stage only from *E*. *hemionus* (IPS83152, Table 1). Therefore, we used superimposition in this equid to establish the relationship between the non-cyclical BGM and birth. Since this non-cyclical feature shares several characteristics between species (position, associated histological changes), we considered this structure analogous in all equids analyzed. This allowed us to extrapolate this life history trait to the other species under study.

We further analyzed bone tissue types and vascularization in the proximity of the non-cyclical BGM to identify any histological changes related to the deposition of this line. Bone tissue types were classified following Francillon-Vieillot et al. [54] and de Margerie et al. [1], while the description of the different components of the fibrolamellar complex (FLC) follows Prondvai et al. [55]. These authors indicate that the FLC is composed of a mix of “fibrous” or woven bone (WB) and “lamellar” or parallel-fibered bone (PFB) [55]. The pattern of vascularization was also classified following classical bibliography [1,54]. In all limb bones studied, we quantitatively analyzed the area of the longitudinal vascular canals (VCs) before and after the presence of the non-cyclical BGM. We restricted the measurements to longitudinal VCs and we did not consider other VC orientations because we have previously noted a difference in size of VCs with this specific arrangement regarding its location in the inner or in the outer cortex of *E*. *hemionus*’ foals [11]. VCs with blurred contours were not drawn or measured (S1 Fig) to reduce error. Secondary osteons (Haversian canals) were also excluded from the analysis (S1 Fig), as the present research is only focused on primary bone. Measurements were taken with Image J software on areas of 2.6 x 2.2 mm (5.7 mm^2^) in the antero-medial region of each bone, at both sides of the non-cyclical BGM (S1 Fig). For each longitudinal VC, we used the circle tool provided by this computer program to manually adjust an ellipse to the edges of this biological structure (S1 Fig). Afterwards, the area of such ellipse was calculated with the “Measure” action integrated in the “Analyze” panel of Image J. Statistical analyses were carried out with Java Gui for R version 1.7–16 [56]. For each bone and *Equus* species, we first calculated the main descriptive statistics (*n*, mean, standard deviation) for the variable “area of the longitudinal VCs” regarding the position of the VC before or after deposition of the non-cyclical BGM (Table 2). Afterwards, we tested the variable for normality within each group (before the non-cyclical BGM and after the non-cyclical BGM) on the different bones (femur, tibia, metacarpus and metatarsus) and species studied (*E*. *hemionus*, *E*. *quagga* and *E*. *grevyi*) using Shapiro-Wilk normality test [57]. Because several data did not follow a normal distribution (*p*-value<0.05), we performed a non-parametric test [57] to statistically evaluate the differences in area of the longitudinal VCs at both sides of the non-cyclical BGM within each bone and species under study. Specifically, Mann-Whitney *U* test (also named Mann-Whitney-Wilcoxon, Wilcoxon rank-sum test or Wilcoxon-Mann-Whitney test) was applied [57] and a *p*-value of *p*<0.05 was considered to be statistically significant.

**Table 2:** Perimeter of *E*. *hemionus*’ perinatal bones and perimeter of the neonatal line (NL) identified in other ontogenetic stages of the same species.

Code	Age	Age group	Femur	Tibia	Metacarpus	Metatarsus
IPS83152	< 3 w.	Perinate	75.08	65.4	60.15	61
IPS83153	5 mo.	Foal	79.21	72.31	60.40	61.65
IPS83154	5 mo.	Foal	82.21	71.47	63.53	-
IPS83151	6 mo.	Foal	85.93	66.64	61.29	60.34
IPS83150	6–12 mo.	Yearling	85.53	68.75	59.05	64.72
IPS83149	6–12 mo.	Yearling	76.89	59.38	57.13	56.04
IPS83155	1–2 y.	Juvenile	-	74.04	-	63.81
IPS83876	4.5 y.	Adult	-	-	60.77	60.04
IPS83877	8 y.	Adult	-	-	56.29	53.09

w.: weeks; mo.: months; y.: years. All measurements are expressed in mm.

## Results

**Fig 1:**
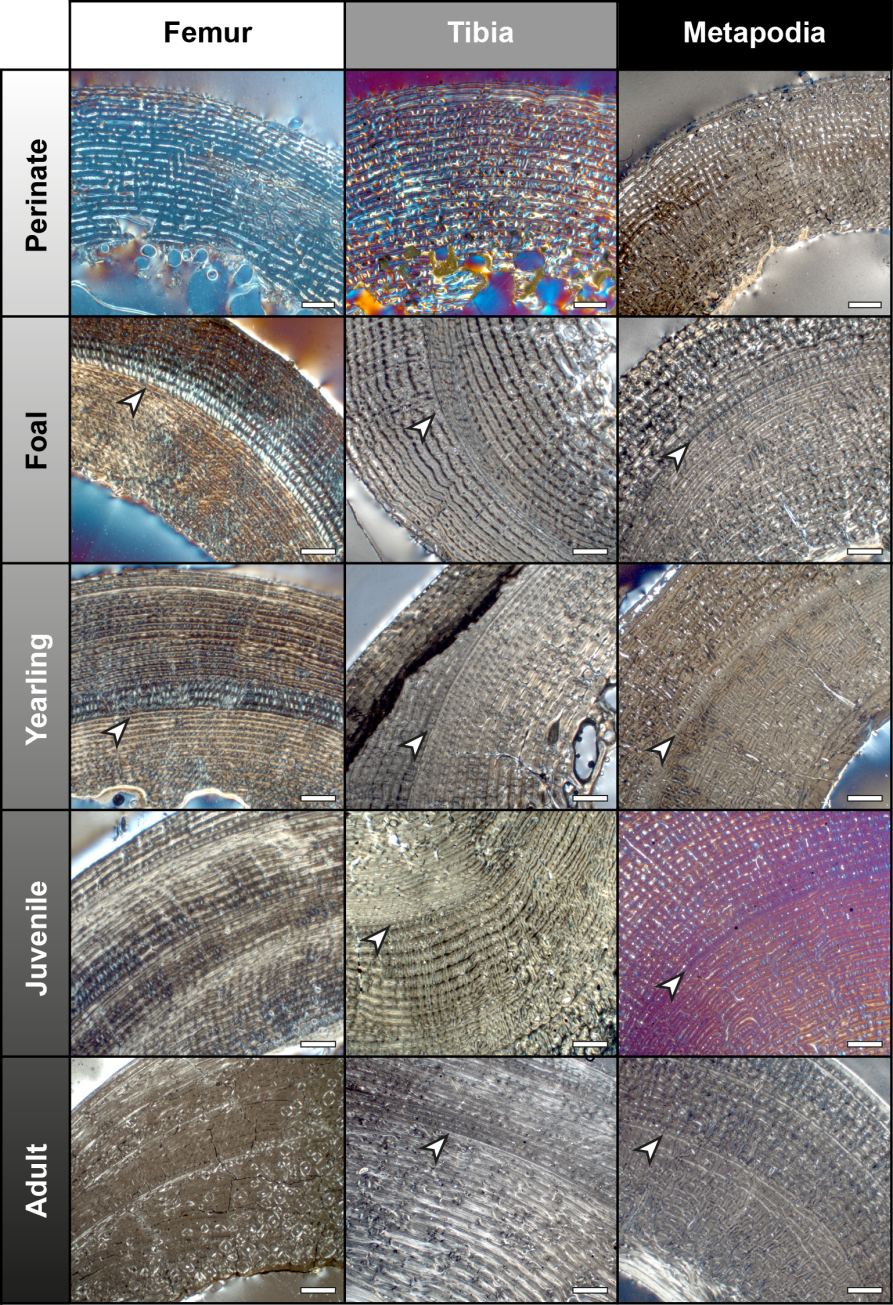
Presence and absence of the neonatal line (NL) in the limb bones of extant *Equus*. From up to down: Femur–IPS92343 (*E*. *quagga*), IPS101798 (*E*. *quagga*), IPS101800 (*E*. *quagga*), IPS101804 (*E*. *grevyi*), IPS83876 (*E*. *hemionus*); Tibia–IPS83152 (*E*. *hemionus*), IPS101798 (*E*. *quagga*), IPS101801 (*E*. *quagga*), IPS101804 (*E*. *grevyi*), IPS84963 (*E*. *grevyi*); Metapodials–IPS83152 (Mc, *E*. *hemionus*), IPS84964 (Mt, *E*. *grevyi*), IPS83150 (Mt, *E*. *hemionus*), IPS83155 (Mc, *E*. *hemionus*), IPS83876 (Mt, *E*. *grevyi*). Mc = metacarpus; Mt = metatarsus. White arrows indicate the NL. Scale bars = 1 mm.

Non-cyclical BGMs are recognized in the bone cortices of all equid species under study, although the identification of these features varies between bones and ontogenetic stages (Fig 1). Only one non-cyclical BGM, which always appears as the first BGM deposited (i.e. the most internal BGM), is found in bone cross-sections (Fig 1). Changes in vascularity and bone matrix typology are also identified associated to the presence of this non-cyclical feature in the different limb bones analyzed.

### Femur

**Fig 2:**
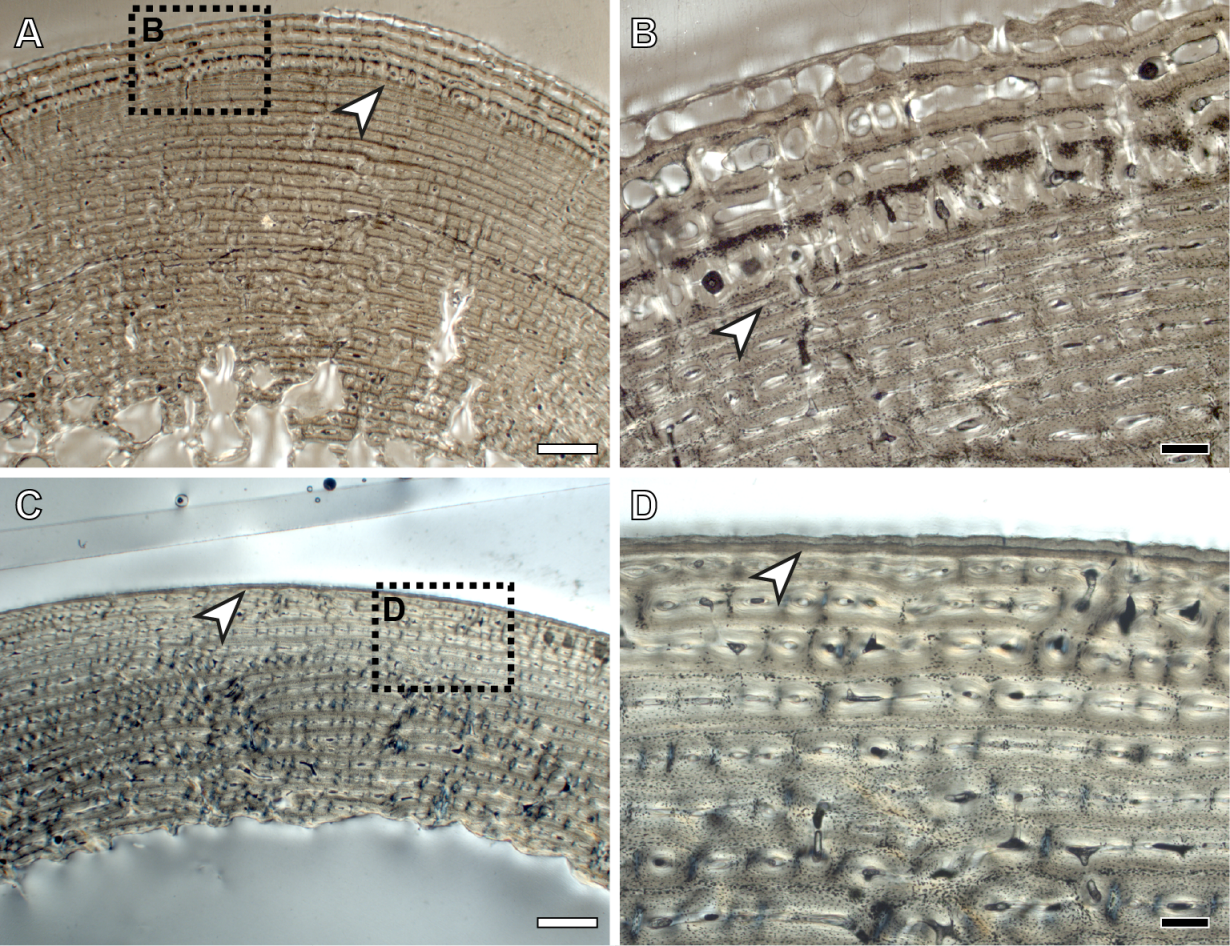
Neonatal line (NL) identified in the femoral and tibial cortex of a 1-month-old Grevy’s zebra. (A) Femoral cortex of IPS101802. (B) Detail of the NL identified in the femur of IPS101802. (C) Tibial cortex of IPS101802. (D) Detail of the NL identified in the tibia of IPS101802. White arrows indicate NL. White dashed rectangles indicate areas of image magnification. White scale bars = 1mm; black scale bars = 200 μm.

**Fig 3:**
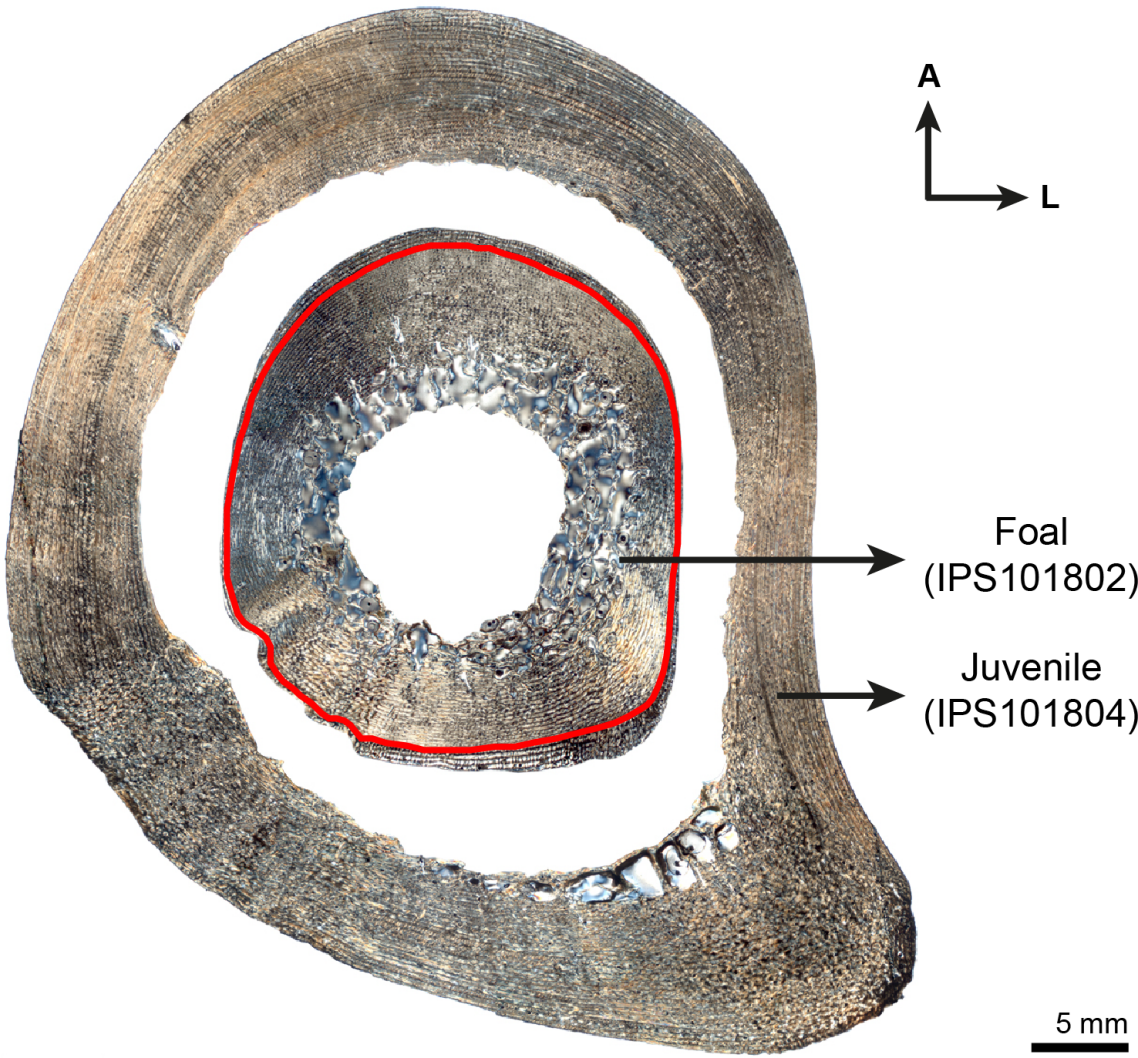
Superimposition of foal’s and juvenile’s femur of *E*. *grevyi*. Figure shows a high resorption of the medullary cavity in the juvenile **(IPS101804)** femur that has erased the neonatal line (NL) identified in the foal **(IPS101802)** (red line). A = anterior, L = lateral.

**Fig 4:**
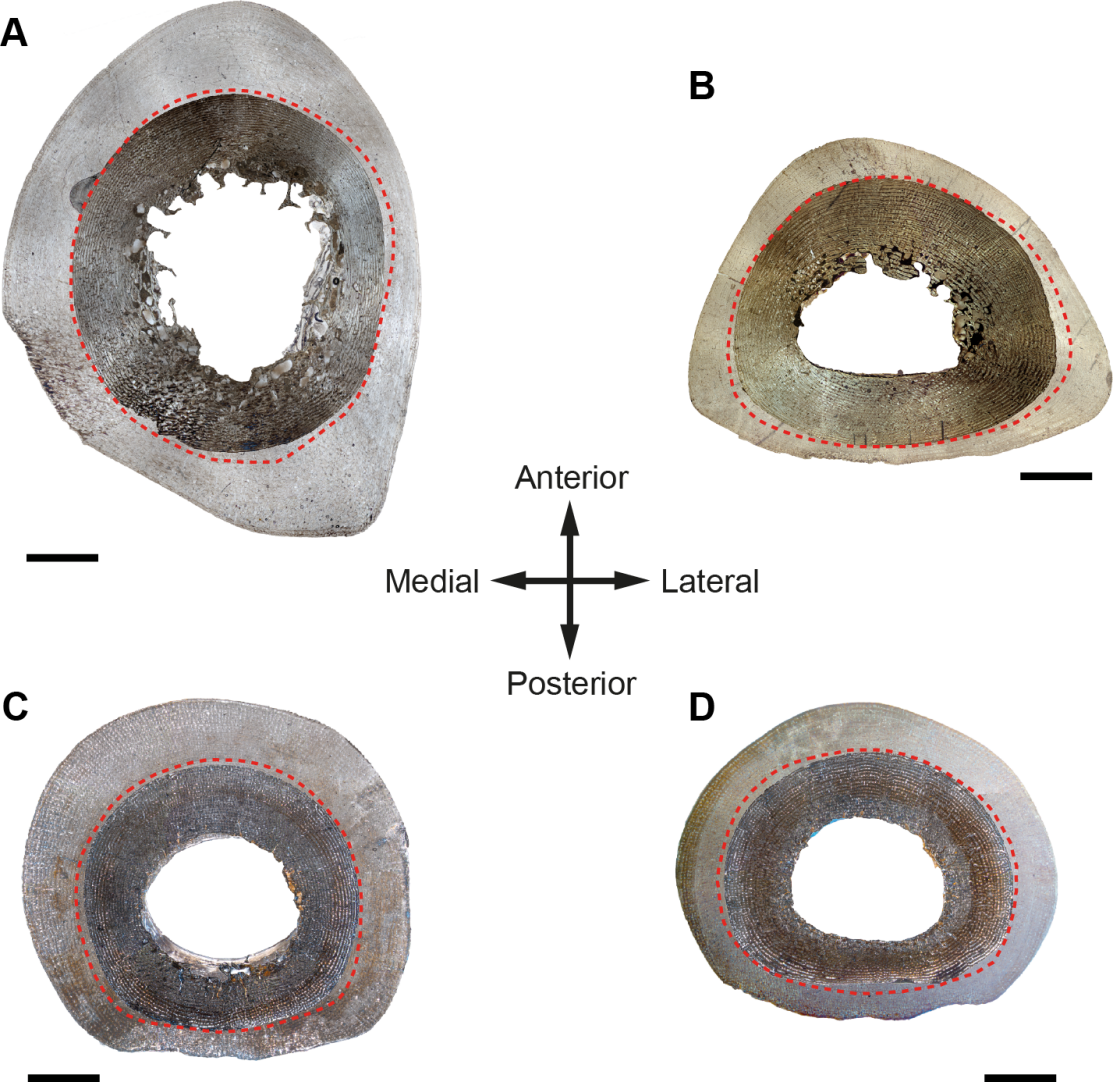
Correspondence between the perimeter of perinatal bones and the neonatal line (NL). Figure shows a match between the perimeter of the perinatal Asiatic wild ass (dark images, IPS83152) and the NL (red dashed line) identified in different limb bones and ontogenetic stages of *E*. *hemionus* (light images). (A) Superimposition of newborn’s and yearling’s (IPS83149) femora. (B) Superimposition of newborn’s and foal’s (IPS83154) tibiae. (C) Superimposition of newborn’s and juvenile’s (IPS83155) metatarsi. (D) Superimposition of newborn’s and adult’s (IPS83876) metacarpi. Scale bars = 5 mm.

In all species analyzed, we identify a non-cyclical BGM in the femoral cortex of foals (IPS83153, IPS83154, IPS83151, IPS101798, IPS92345, IPS92342, IPS101802, IPS84964) and yearlings (IPS83149, IPS83150, IPS92341, IPS101800) (Fig 1). In the youngest animal of our sample, a 1-month-old Grevy’s zebra (IPS101802, Table 1), this feature is situated in the outermost cortex (Fig 2A and 2B). We do not find any non-cyclical BGM, however, in the femur of perinates (IPS83152, IPS92343, IPS92344), juveniles (IPS83155, IPS101804) or adults (IPS83876, IPS83877, IPS92346, IPS84963) (Fig 1). Superimposition performed in this bone shows that the bone tissue formed during the earliest ontogenetic stages (perinatal, foal) is lost in juvenile individuals through resorption of the medullary cavity (Fig 3), which erased any early non-cyclical BGM in the femora of juvenile and/or adult specimens. Interestingly, superimposition performed in the Asiatic wild ass reveals that the cortical perimeter of the perinatal individual almost matches the non-cyclical BGM found in the femora of foals and yearlings (Table 2 and Fig 4A). As shown in Table 2, the perimeter of the perinatal femur is 75.08 mm while the perimeter of the non-cyclical BGM varies between 76.89 mm and 85.93 mm in this bone. For this reason, we consider this BGM as a stress line related to the birth event of the animal and, henceforward, we will refer to it as neonatal line (NL).

**Fig 5:**
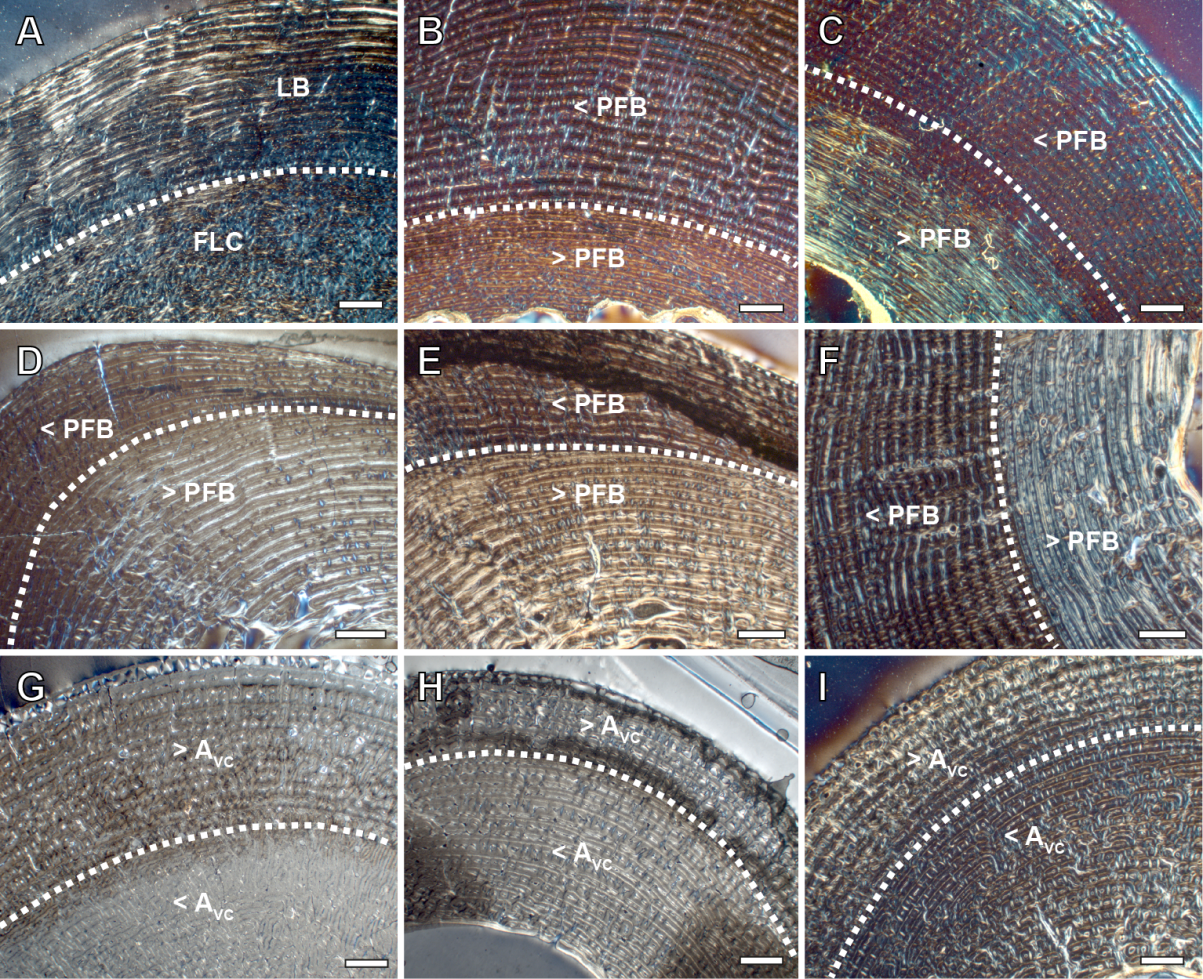
Histological changes associated to the presence of the neonatal line in extant *Equus*. (A) Femoral cortex of *E*. *hemionus* IPS83153. (B) Femoral cortex of *E*. *quagga* IPS92345. (C) Femoral cortex of *E*. *grevyi* IPS84964. (D) Tibial cortex of *E*. *hemionus* IPS83154. (E) Tibial cortex of *E*. *quagga* IPS101801. (F) Tibial cortex of *E*. *grevyi* IPS101804. (G) Metacarpal cortex of *E*. *hemionus* IPS83150. (H) Metatarsal cortex of *E*. *quagga* IPS104356. (I) Metatarsal cortex of *E*. *grevyi* IPS84964. White dotted line indicates the neonatal line. A_VC_ = area of the longitudinal VCs; FLC = fibrolamellar complex; LB = laminar bone; PFB = parallel-fibered bone. Scale bars = 1 mm.

**Fig 6:**
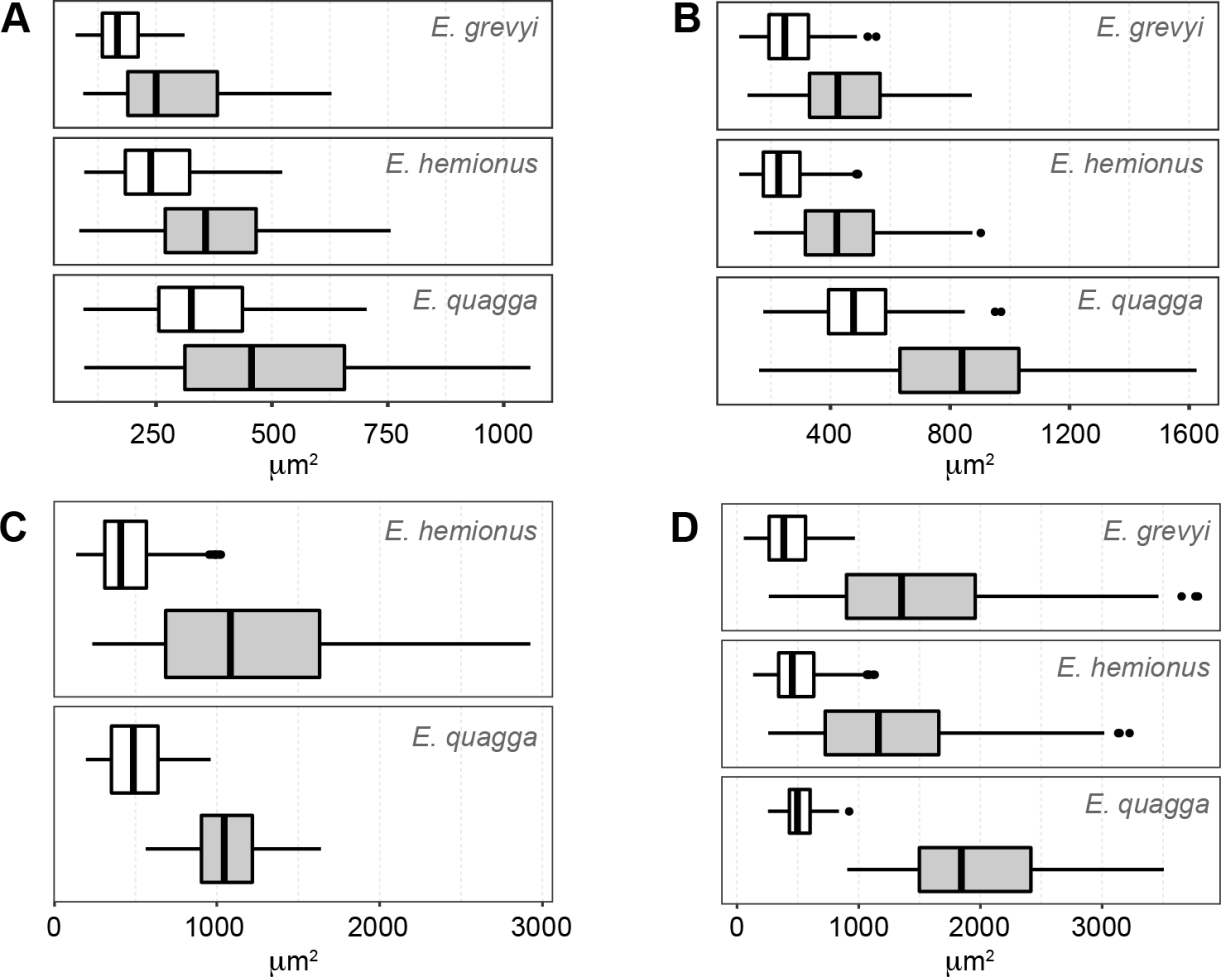
Area of the vascular canals (VCs) at both sides of the neonatal line (NL). (A) Femora. (B) Tibiae. (C) Metacarpi. (D) Metatarsi. White boxplot = Area of the VCs located before the deposition of the NL; grey boxplot = Area of the VCs located after the deposition of the NL.

**Fig 7:**
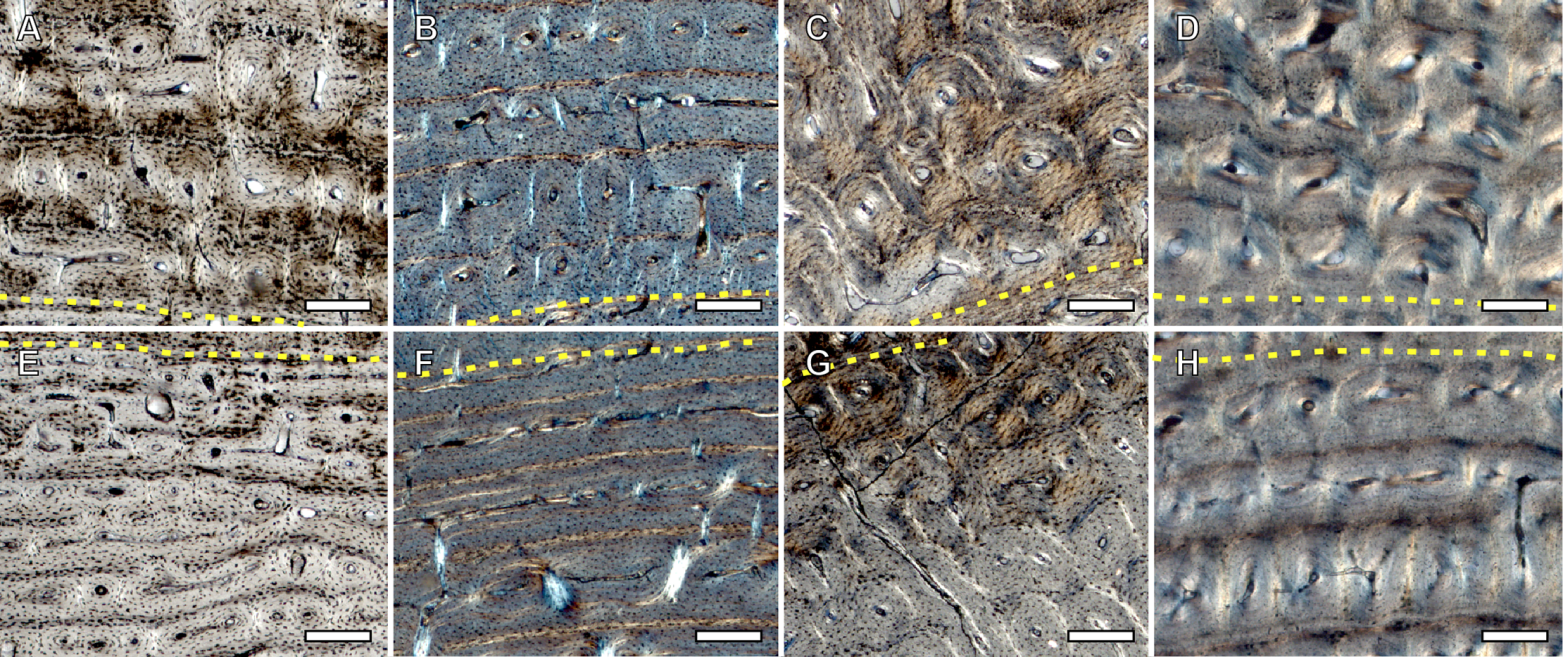
Longitudinal vascular canals (VCs) formed after and before the neonatal line (NL) (yellow dotted line). (A) VCs formed after deposition of the NL in the femur of *E*. *quagga* IPS92345. (B) VCs formed after deposition of the NL in the tibia of *E*. *hemionus* IPS83153. (C) VCs formed after deposition of the NL in the metacarpus of *E*. *hemionus* IPS83150. (D) VCs formed after deposition of the NL in the metatarsus of *E*. *grevyi* IPS84964. (E) VCs formed before deposition of the NL in the femur of *E*. *quagga* IPS92345. (F) VCs formed before deposition of the NL in the tibia of *E*. *hemionus* IPS83153. (G) VCs formed before deposition of the NL in the metacarpus of *E*. *hemionus* IPS83150. (H) VCs formed before deposition of the NL in the metatarsus of *E*. *grevyi* IPS84964. Scale bars = 200 μm.

We recognize several histological changes related to bone tissue type and to the arrangement of the VCs in the surroundings of the NL (Fig 5A–5C). In femora of *E*. *hemionus*, the NL divides the bone cortex in two areas: an internal one composed of FLC with longitudinal VCs and an external ring of laminar bone (Fig 5A). In *E*. *quagga* and *E*. *grevyi*, however, the NL is associated with a change in the components of the FLC, as femora of both zebras shows a higher proportion of PFB before the deposition of this non-cyclical BGM (Fig 5B and 5C). In all species studied, we observe significant differences in the area of the longitudinal VCs before and after the NL (*E*. *hemionus*, W = 14004.5 and *p*<0.001; *E*. *quagga*, W = 9408.5 and *p*<0.001; *E*. *grevyi*, W = 3208 and *p*<0.001). VCs identified before this mark are smaller than those found after this feature in equid femoral cortices (Figs 6A and 7A–7E). Specifically, longitudinal VCs formed before deposition of the NL present a mean area of ≈250 μm^2^, ≈350 μm^2^ and ≈170 μm^2^ in the Asiatic wild ass, the Plains zebra and the Grevy’s zebra respectively (Table 3 and Fig 6A). On the contrary, the mean area of the VCs found after the NL is ≈370 μm^2^ in *E*. *hemionus*, ≈500 μm^2^ in *E*. *quagga* and ≈290 μm^2^ in *E*. *grevyi* (Table 3 and Fig 6A).

**Table 3:** Area of the longitudinal vascular canals (VCs) at both sides of the neonatal line in the limb bones of *Equus*.

	**FEMORA**				**TIBIAE**			
	**Before the NL**		**After the NL**		**Before the NL**		**After the NL**	
	**n**	**Mean ± SD**	**n**	**Mean ± SD**	**n**	**Mean ± SD**	**n**	**Mean ± SD**
***E*. *hemionus***	137	254.8**±**95.2	135	372.4**±**137.6	150	243.5**±**91.6	169	446.8**±**177
***E*. *quagga***	123	356.6**±**142.4	114	501.3**±**246.1	156	497.6**±**146.4	147	823.3**±**301.3
***E*. *grevyi***	59	177**±**57.7	70	292.5**±**137.3	74	270.7**±**100.6	128	458**±**170
	**METACARPI**				**METATARSI**			
	**Before the NL**		**After the NL**		**Before the NL**		**After the NL**	
	**n**	**Mean ± SD**	**n**	**Mean ± SD**	**n**	**Mean ± SD**	**n**	**Mean ± SD**
***E*. *hemionus***	449	455.4**±**191	454	1213.7**±**660.6	514	499.1**±**209.2	427	1284.4**±**655.7
***E*. *quagga***	72	508.8**±**184.5	29	1080.3**±**276.1	52	525.4**±**155.9	34	1962.9**±**656.1
***E*. *grevyi***	-	-	-	-	100	426.1**±**202.4	72	1502.6**±**873

n = number of observations; SD = standard deviation.

### Tibia

From foals to adults, we find a non-cyclical BGM in the tibiae of all species under study (IPS83153, IPS83154, IPS83151, IPS83150, IPS83149, IPS83155, IPS83876, IPS83877, IPS101798, IPS104356, IPS104357, IPS101801, IPS101802, IPS84964, IPS101804, IPS84963) (Fig 1). The same as in the femur, this mark is identified in the tibial cortex of IPS101802 (Fig 2C and 2D), the youngest specimen of *E*. *grevyi* aged one month old (Table 1). Only the perinatal tibia of *E*. *hemionus* (IPS93152) does not show a non-cyclical BGM in its cortex (Fig 1). Superimposition of individuals performed with the tibiae of *E*. *hemionus* indicates a correspondence between the perimeter of the non-cyclical BGM and that of the perinatal individual (Fig 4B), as the perimeter of the NL measures between ≈66 mm and ≈74 mm and the perimeter of IPS83152 measures 65.4 mm (Table 2). This also indicates that the non-cyclical feature is recording the moment of birth in this bone.

Regarding bone tissue, tibial cortices of *E*. *hemionus*, *E*. *quagga* and *E*. *grevyi* show a change in the proportions of the FLC components associated to the deposition of the neonatal line (Fig 5D–5F). In all samples, we notice that the FLC formed before the deposition of the NL presents a higher proportion of PFB than the FLC deposited after the presence of the NL (Fig 5D–5F). The area of the longitudinal VCs also varies significantly at both sides of the NL in all species under study (*E*. *hemionus*, W = 21609.5 and *p*<0.001; *E*. *quagga*, W = 19060 and *p*<0.001; *E*. *grevyi*, W = 7903.5 and *p*<0.001). The VCs identified before this feature are smaller than those deposited after the NL (Figs 6B and 7B and 7F). The mean area of the longitudinal VCs found earlier than the presence of the NL is ≈240 μm^2^ in *E*. *hemionus*, ≈350 μm^2^ in *E*. *quagga* and ≈170 μm^2^ in *E*. *grevyi* (Table 3 and Fig 6B). After this feature, longitudinal VCs present a mean area of ≈440 μm^2^, ≈820 μm^2^ and ≈460 μm^2^ respectively (Table 3 and Fig 6B).

### Metapodial bones

We recognize a non-cyclical BGM in metacarpus and metatarsus of foals (IPS83153, IPS83154, IPS83151, IPS104356, IPS84964), yearlings (IPS83150, IPS83149), juvenile (IPS83155) and adults (IPS83876, IPS83877, IPS84963) of all *Equus* species examined (Fig 1). Thus, the only ontogenetic stage that does not record this mark is the perinatal period (IPS83152) (Fig 1). The youngest individual of our sample that presents this non-cyclical BGM is a 4-month-old Grevyi’s zebra (IPS84964, Table 1). In this specimen, this mark is identified in the middle cortex of the metatarsus (Fig 1). Superimposition of individuals in the Asiatic wild ass shows that the perimeter of the non-cyclical BGM identified in both metapodials is almost the same as the perimeter of the perinatal kulan (Fig 4C and 4D), which supports the hypothesis that this mark is deposited around birth. The perimeter of *E*. *hemionus*’ perinatal metapodia measures ≈60 mm, while the perimeter of the non-cyclical BGM identified in older ontogenetic stages is ≈53–64 mm in these bones (Table 2).

We do not find differences in bone tissue type or in the arrangement of VCs associated to the presence of the NL, although we also observe a variation in the size of the longitudinal VCs at both sides of this feature in metacarpi and metatarsi of the different *Equus* species studied (Fig 5G–5I). In our equid metapodial sample, VCs found earlier than the deposition of the NL are significantly smaller than those identified after the mark (Metacarpi: *E*. *hemionus*, W = 181501 and *p*<0.001; *E*. *quagga*, W = 2017 and *p*<0.001; Metatarsi: *E*. *hemionus*, W = 196227 and *p*<0.001; *E*. *quagga*, W = 1767 and *p*<0.001; *E*. *grevyi*, W = 6677 and *p*<0.001) (Table 3 and Figs 6C and 6D and 7C and 7D and 7G and 7H). The mean area of the inner VCs is 400–500 μm^2^ (Table 3 and Fig 6C and 6D), while it is 1000–2000 μm^2^ (Table 3 and Fig 6C and 6D) for the VCs found in the outer cortex of both metapodial bones.

## Discussion

Most of the skeletochronological research developed so far focuses on the study of cyclical bone growth marks present in the limb bones of vertebrates [20,22,24]. Hitherto, however, little is known about the causes leading to the deposition of non-cyclical features. Non-cyclical BGMs identified in bone cortex are usually classified as cortical drift lines [20], while non-cyclical lines that record periods of physiological stress are less studied [23]. The non-cyclical BGM identified in our equid sample is not restricted to a specific location within the cross-section of the bone, but it can be followed around the whole cortex (Fig 3). Thus, it cannot be considered a resorption line caused by cortical drift [20]. Rather, it appears to record a specific biological event: the moment of birth. The NL is well described in dental histology and can be easily found in the enamel and dentine of different mammalian species [58–62]. To our knowledge, however, there is no previous reference to the NL in histological studies of any mammalian taxon. With the present study, hence, we provide the first description of a non-cyclical BGM related to the moment of birth in the limb bones of mammals. From foals to adults, we recognized a NL in all species and bones studied, except for the femora of juvenile and adult specimens (Fig 1), where it has been removed during resorption of the medullary cavity (Fig 3). Also, we did not identify a NL in any of the perinatal bones analyzed (Fig 1), probably indicating that the youngest animals of our sample died before or at birth (stillborn). The identification of the NL is essential for histological research performed in the appendicular bones of mammals, and especially in Equidae. For instance, in a previous study on the limb bone histology of *Hipparion concudense*, Martínez-Maza et al. [63] reported the presence of a non-cyclical BGM that these authors interpreted as a drift line. Based on their descriptions and the images provided by the authors [63], however, we consider this non-cyclical feature as a NL. Though the identification of the NL does not affect the conclusions of their study, it provides a time anchorage (the moment of birth) for skeletochronological studies. It further facilitates the estimation of the size at birth in extinct species, an important life history trait in mammals [64]. Considering that the NL represents the bone circumference at the moment of birth, its dimension can be used to estimate the weight [65] of the newborn foal, a proxy of its body size [66].

Despite the huge amount of studies that describe hatching lines and metamorphosis lines in reptiles and amphibians [27–38], only few studies report the presence of non-cyclical BGMs related to life history events in mammalian bones [5,39]. In their study of *Microcebus murinus*, Castanet et al. [5] described a weak mark in several long bones of this species that they suggest might be recording the weaning event. Likewise, Morris [39] identified a line of weaning in the jaws of the hedgehog. In the equid species analyzed here, weaning occurs at 1–1.5 years (Asiatic wild ass), around the first year (Plains zebra) and at the eighth month of life (Grevy’s zebra) [40,41,67,68]. However, we found the non-cyclical BGM in our specimens at an earlier age (Fig 1), already present only one month after birth in the youngest foal of *E*. *grevyi* (Fig 2). Thus, our results do not match the weaning event previously described for the prosimian *Microcebus* or the hedgehog [5,39]. The coincidence between the perimeter of the perinatal individual and that of the non-cyclical BGM identified in other age groups of *E*. *hemionus* (Table 2 and Fig 4) supports however the idea that this feature is related to the birth of the animal. In equids, as well as in other groups of mammals, birth is a moment of physiological stress where many organic systems of the foal (respiratory, circulatory, etc.) undergo important changes that adapt them to a life *ex utero* [69]. This transition also implies a variation in the concentration values of the most important hormones [69–71] that regulate bone growth [72]. At the moment of birth, equid foals present low concentration values of growth and thyroid hormones [70,71], endocrine regulators that control several key growth factor signaling pathways to tune and stimulate skeletal growth [72,73]. At this moment, neonatal foals also show high levels of cortisol [69], a glucocorticoid that constrains bone growth in stressful conditions [72]. This hormonal scenario, hence, suggests an arrest of bone growth at the moment of birth, which, in turn, agrees with the presence of a rest line [23,54] such as the NL found in our sample (Fig 1).

We have also identified changes in bone tissue type and in vascularization in the surroundings of the NL (Fig 5). Regarding bone matrix, our study reveals a notable change in the proportion of PFB within the FLC shortly before deposition of the NL in the femora of both zebras and in the tibiae of all *Equus* species analyzed (Fig 5B–5F). Previous studies have also reported a change in bone tissue type associated to a non-cyclical BGM that reflect a biological event, such as the hatching line [29,30,74]. Contrary to our observations in equids, however, dinosaurs and reptiles show a change from a disorganized bone matrix (woven-fibered bone) before the hatching line to a more organized one (parallel-fibered bone) after this line [29,30,74]. The differences between these findings and our results from *Equus* might be related to the higher postnatal growth rates of mammals in comparison with that of reptiles [75–77]. In fact, histological studies on Elephantidae [78] and thoroughbred horses [79] agree with our results, as the postnatally deposited FLC in these animals also presents a lower proportion of PFB than the prenatally formed bone tissue [78,79]. In agreement with these investigations [78,79], the reduction in PFB observed in the present study (Fig 5B–5F) suggests a higher rate of bone deposition in *Equus* after birth. Nonetheless, the qualitative variation in PFB observed in equids (Fig 5B–5F) can also been interpreted as an adaptation to the change in biomechanical loads [25,26] that these animals experience after birth. In this sense, it has been suggested that the disposition of VCs within the bone tissue might be related to biomechanics [80,81]. The change in the orientation of the VCs observed in *E*. *hemionus* just after deposition of the NL (Fig 5A), thus, seems to be associated with the onset of weight-bearing and locomotion just after birth [7]. In our equid sample, we also observe a change in diameter of the longitudinal VCs associated to the deposition of the NL (Figs 5G–5I and 7). In all limb bones and species examined, longitudinal VCs identified before this feature are significantly smaller than those found after the mark (Table 3 and Figs 6 and 7). In metapodia, the mean area of the VCs formed after deposition of the NL even doubles the mean area of those VCs formed before the formation of the NL (Table 3 and Fig 6). These results agree with the findings of Stover et al. [79] of perinatal histological modifications in thoroughbred horses. Although these authors analyzed primary osteons of the third metacarpus instead of VCs, they described that postnatally formed primary osteons are larger than those formed prenatally [79]. Larger VCs have also been related to higher rates of bone deposition [1]. Thus, the higher area of the VCs identified after deposition of the NL in *Equus* limb bones (Table 3 and Fig 6) also suggests higher rates of postnatal bone formation in equids.

## Conclusions

In the present research, we describe for the first time a non-cyclical BGM in the limb bones of mammals that records the moment of birth. During this life history event, physiological levels of cortisol, thyroid hormones, and growth hormones agree with a period of growth arrest in the newborn foal that leads to the deposition of the NL. We identified this NL in femora, tibiae and metapodia of *E*. *hemionus*, *E*. *quagga* and *E*. *grevyi*. While the NL is observable in tibia, metacarpus and metatarsus of foals, yearlings, juveniles and adults, it disappears in juvenile and adult femora due to early resorption of the medullary cavity in this bone. We have also identified several histological changes associated to the presence of the NL. On the one hand, the proportion of PFB within the FLC is reduced after deposition of the NL in the femora of plains and Grevyi’s zebra and in the tibiae of all *Equus* species studied. This suggests a higher rate of bone deposition in these bones after birth. In the femora of the Asiatic wild ass, however, we observed a change in orientation of the VCs from a preferentially longitudinal to a circular organization, which is likely related to biomechanical loads. Finally, a difference in size of the longitudinal VCs has been identified in all limb bones and species studied, showing larger VCs after deposition of the NL. Our findings about perinatal bone histology, both the presence of the NL and the histological changes (bone tissue and vascularization) associated to it, are essential for future skeletochronological studies in equids and related mammals.

## Supporting information

S1 Fig Methodology employed to estimate the area of the longitudinal vascular canals (VCs) in the limb bones of *Equus* at both sides of the NL. (A) Metatarsal cross-section of *E*. *hemionus* IPS83149. White dashed rectangles indicate areas of image magnification. (B) VCs after the presence of the NL in *E*. *hemionus* IPS83149. (C) VCs before the presence of the NL in *E*. *hemionus* IPS83149. For each VC, we adjusted an ellipse (red circles) and measured its area with ImageJ software. Secondary osteons (white star), canals with no longitudinal orientation and non-circular form (green star) or canals with blurred edges (blue star) were not measured. Yellow dashed line indicates the NL. White scale bar = 200 μm.
(TIF)
