# Supplementary figures and images for: Limb bone histology records birth in mammals
Source: PLoS One. 2018 Jun 20;13(6):e0198511. doi: 10.1371/journal.pone.0198511 (PMC6010216; doi:10.1371/journal.pone.0198511)

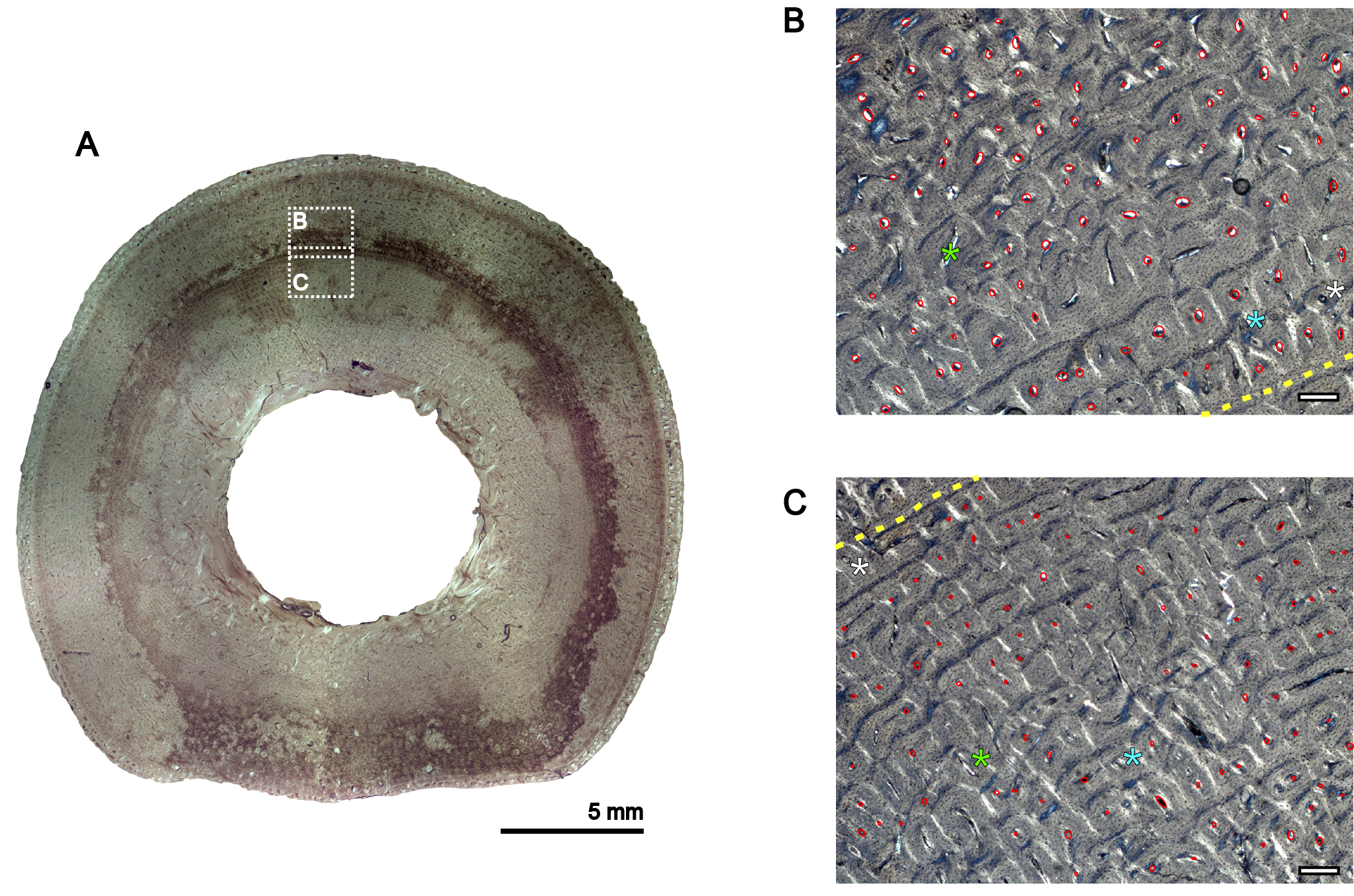

Supplement: S1 Fig — (A) Metatarsal cross-section of E. hemionus IPS83149. White dashed rectangles indicate areas of image magnification. (B) VCs after the presence of the NL in E. hemionus IPS83149. (C) VCs before the presence of the NL in E. hemionus IPS83149. For each VC, we adjusted an ellipse (red circles) and measured its area with ImageJ software. Secondary osteons (white star), canals with no longitudinal orientation and non-circular form (green star) or canals with blurred edges (blue star) were not measured. Yellow dashed line indicates the NL. White scale bar = 200 μm. (TIF) [file pone.0198511.s001.tif]
